# Effect of the Structural Modification of Plant Proteins as Microencapsulating Agents of Bioactive Compounds from Annatto Seeds (*Bixa orellana* L.)

**DOI:** 10.3390/foods13152345

**Published:** 2024-07-25

**Authors:** Julián Quintero Quiroz, Víctor Velazquez, Juan D. Torres, Gelmy Ciro Gomez, Efren Delgado, John Rojas

**Affiliations:** 1 Faculty of Ciencias de la Nutrición y los Alimentos, CES University, Calle 10 # 22-04, Medellin 050018, Colombia; 2 Department of Ciencias Farmacéuticas y Alimentarias, University of Antioquia, Calle 67 No. 53-108, University Campus, Medellín 050010, Colombia; juan.torreso@udea.edu (J.D.T.); gelmy.ciro@udea.edu.co (G.C.G.); jhon.rojas@udea.edu.co (J.R.); 3 Department of Family and Consumer Sciences, College of Agriculture, Consumer and Environmental Sciences, New Mexico State University, NMSU Gerald Thomas Hall Room, 308 P.O. Box 30003 MSC 3470, Las Cruces, NM 88003, USA; yambe@nmsu.edu

**Keywords:** plants proteins, modification proteins, antioxidant

## Abstract

This project studied the use of lentil protein (LP) and quinoa protein (QP) in their native and modified states as carrier material in the encapsulation process by the ionic gelation technique of annatto seed extract. Soy protein (SP) was used as a model of carrier material and encapsulated bioactive compounds, respectively. The plant proteins were modified by enzymatic hydrolysis, N acylation, and N-cationization to improve their encapsulating properties. Additionally, the secondary structure, differential scanning calorimetry (DSC), solubility as a function of pH, isoelectric point (pI), molecular weight (MW), the content of free thiol groups (SH), the absorption capacity of water (WHC) and fat (FAC), emulsifier activity (EAI), emulsifier stability (ESI), and gelation temperature (Tg) were assessed on proteins in native and modified states. The results obtained demonstrated that in a native state, LP (80.52% and 63.82%) showed higher encapsulation efficiency than QP (73.63% and 45.77%), both for the hydrophilic dye and for the annatto extract. Structural modifications on proteins improve some functional properties, such as solubility, WHC, FAC, EAI, and ESI. However, enzymatic hydrolysis on the proteins decreased the gels’ formation, the annatto extract’s encapsulated efficiency, and the hydrophilic dye by the ionic gelation method. On the other hand, the modifications of N-acylation and N-cationization increased but did not generate statistically significant differences (*p*-value > 0.05) in the encapsulation efficiency of both the annatto extract and the hydrophilic dye compared to those obtained with native proteins. This research contributes to understanding how plant proteins (LP and QP) can be modified to enhance their encapsulating and solubility properties. The better encapsulation of bioactive compounds (like annatto extract) can improve product self-life, potentially benefiting the development of functional ingredients for the food industry.

## 1. Introduction

The use of plant proteins has increased significantly in recent years. Proteins from plant sources are considered isolated macromolecules from highly renewable and abundant sources; their uses and potentization are among current market trends, and they are gaining popularity as a non-allergenic source of protein that offers a clean label to food products [1,2].

These proteins can be used as functional ingredients, as they have been proven to reduce the risk of cardiovascular disease and blood pressure [3]. In addition to their nutritional contribution, the flexible, amphiphilic, amphiprotic, and dynamic protein structure according to the conditions of the environment give proteins functional properties, such as the capacity to modify the rheological properties and surface tension of the medium, to stabilize colloidal systems, as well as the absorption capacity of water and fat, the ability to form gels, and the capacity to encapsulate bioactive compounds by techniques such as spray drying, coacervation, and ionic gelation [4,5,6]. Isolated soy proteins have been studied more frequently than other proteins from plant sources, possibly due to their wide commercial availability [5]. However, some research has used protein isolates from lentils, sunflower seeds, quinoa, peas, and beans as emulsion stabilizers, gelling agents, and carrier material in the encapsulation processes of α-tocopherol [7], flaxseed oil [3], probiotic bacteria [8], and vitamin D [6,9].

The annatto seed extract has a high content of bixin and seven polyphenol compounds, such as catechin, chlorogenic acid, chrysin, butein, hypolaetin, licochalcone A, and xanthohumol [10]. These bioactive compounds can deactivate sensitizers’ excited triplet state, which is usually associated with photosensitization to quench singlet oxygen, sweeping free radicals, and the denaturation of proteins from cell membranes [11,12,13]. These characteristics give annatto seed extracts the ability to be antioxidant and antimicrobial [13,14]. As stated in the literature, these bioactive compounds are not stable under conditions such as extreme pH, light, and high temperatures, which leads to the enforcement of techniques such as encapsulation to increase its stability and enhance its applications in the food, pharmaceutical, and cosmetic industries [13].

Our recent research suggests that lentil (LP) and quinoa (QP) protein isolates represent a highly suitable carrier material for the encapsulation process of annatto seed extract by ionic gelation, with encapsulation efficiencies of 68.61% and 58.38% for LP and QP, respectively [15]. Regarding the stability of the bioactive compounds, both proteins protect and stabilize the extract’s antioxidant and antimicrobial activities, even up to a storage temperature of 65 °C for 12 d [15].

Encapsulation technology plays a crucial role in enhancing the stability, bioavailability, and targeted delivery of bioactive compounds in food. By entrapping these compounds within protective matrices, encapsulation shields them from adverse environmental conditions (such as temperature and oxygen) and physiological factors (such as gastric acid). Among the various coating materials used, proteins—such as whey proteins, casein, and soy proteins—stand out as effective encapsulation agents [15]. These proteins form stable microcarriers or nanoparticles that can modulate release kinetics and protect sensitive ingredients during food processing and storage. Additionally, protein-based encapsulation allows for tailored release profiles, ensuring optimal bioactivity. Overall, understanding the impact of different encapsulation materials on bioactive compound release is essential for advancing functional foods and nutraceuticals.

However, the application of proteins from plant sources in the encapsulation processes of active compounds remains very limited, mainly due to their low solubility in water and the need for high concentrations that are required to generate a change in the viscoelastic properties of the dispersions. For this reason, in several studies, the structural modification of proteins has been proposed as a strategy to generate changes in their functional properties, allowing them to increase their solubility in water, emulsifying capacity, and capacity for gel formation [16,17,18,19,20]. These properties can increase the encapsulation efficiencies of modified proteins to potentiate their use as coating materials in the encapsulation processes of active compounds. Some of the most outstanding research studies were carried out by Nesterenk et al. in 2012 [21]. They evaluated the effect of enzymatic hydrolysis, the treatment of N-acylation, and N-cationization of isolated soy proteins on their functional properties, specifically, on the encapsulation capacity of α-tocopherol, using the spray drying method [21]. For the report, the structural changes produced by the three methodologies, with a more significant effect of N-acylation, allowed for decreasing the drop size and modifying the viscoelastic properties of the emulsions produced during the encapsulation process. As a result, an increase was obtained in the encapsulation efficiency of the bioactive compounds from 80% to 87% [21].

Likewise, other researchers have evaluated the effect of enzymatic hydrolysis, N-acylation, and N-cationization on the functional properties of proteins. Adler-Nissen and Olsen observed that the emulsifying and foaming properties of soy protein could be improved by enzymatic hydrolysis to a limited degree of hydrolysis of up to 10%, and the peptides obtained must be large enough to form a stable film around the droplets of the dispersed phase [22]. Meanwhile, the use of modifications such as N-acylation, covalently linking a fatty chain in the protein structure, and N-cationization implies the generation of positive charges in the molecules by grafting cationic groups. Little explored methodologies have been used to increase the functional properties of proteins [18,23]. This study aimed to (i) evaluate the effect of structural modifications by enzymatic hydrolysis, N-acylation, and N-cationization of the LP and QP on their functional properties and (ii) compare the encapsulation efficiency of annatto seed extract by ionic gelation employed as the carrier material of LP and QP in native and modified states.

## 2. Materials and Methods

### 2.1. Samples

Soy protein (SP) isolates used as controllers were purchased from Bell Chem International S.A.S (Medellín, Colombia). The quinoa (*Chenopodium quinoa*) and lentil (*Lens culinaris*) seeds were purchased from a farmer’s market in Medellín. Annatto seeds were donated by a production farm located in Córdoba, Colombia.

### 2.2. Chemicals

Dithiothreitol (DTT), glutaraldehyde, o-phthaldialdehyde (OPA), sodium carbonate, 5.5-dithio-bis-(2-nitrobenzoic acid) (DTNB), sodium acetate trihydrate, concentrated hydrochloric acid, absolute ethanol, sodium citrate, acetone, and bixin standard were purchased from Sigma-Aldrich (St. Louis, MO, USA). Ethylenediaminetetraacetic acid, calcium chloride, sodium dodecyl sulfate, and glycerin were purchased from Merck KGaA (Darmstadt, Germany). Tris-HCL molecular biology grade was obtained from Becton Dickinson (Franklin Lakes, NJ, USA). Acrylamide, bis-acrylamide, and Coomassie blue were purchased from Bio-Rad Laboratories (Berkeley, CA, USA). Finally, Alcalase 2.4L and ammonium sulfate were purchased from Novozymes (Medellín, Colombia) and Bell Chem International (Medellín, Colombia), respectively.

### 2.3. Extraction of Proteins from Quinoa and Lentils

The quinoa and lentil seeds were oven-dried (model IMP180, Thermo Fisher Scientific, Waltham, MA, USA) at 37 °C for 48 h, followed by milling; they were passed through a number 60 mesh (250 µm size) and were stored in desiccators until use. Proteins were extracted from the powder of the seeds by leaching at constant agitation for 12 h. Both extractions were performed with a 0.1 M Tris-HCl buffer at a pH of 10 at 25 °C at a buffer-to-plant material ratio of 1:10 and 1:5 (*v*:*w*) for LP and QP, respectively. Afterward, the supernatant was separated from the solid to precipitate the extracted proteins using ammonium sulfate at a saturation of 100%. Finally, the solution was centrifuged at 12,000× *g* for 10 min (Hermle Z206A, HERMLE Labortechnik GmbH, Wehingen, Germany) followed by dialysis using a 3 kDa cellulose membrane (Fisherbrand™, Sunnyvale, CA, USA) for two days against distilled water (Type II water) [24]. The obtained proteins were freeze-dried and stored in desiccators until use. Protein content was assessed according to the AOAC (Association of Official Analytical Chemists) methods (AOAC 2011.04) [25], resulting in 35.0 ± 2.03% and 41.4 ± 2.98% (N × 5.3) for quinoa and lentils, respectively.

### 2.4. Protein Structural Modification

The degree of structural modification of the proteins was determined by the variation in amino groups (NH_2_) during the modification. Enzymatic hydrolysis, N-acylation, and N-cationization techniques were used for structurally modified proteins.

#### 2.4.1. Quantification of Free Amino Groups (NH_2_)

Free amino groups from the proteins were determined by the o-phthaldialdehyde method described by Nielsen, Petersen, and Dambmann (2001) [26], which measures the reaction between the free amino group (NH_2_), o-phthaldialdehyde (OPA), and dithiothreitol (DTT). For an OPA reagent, 160 mg of OPA was dissolved in 4 mL of ethanol and mixed with 150 mL of a solution with 7.62 g of decahydrate sodium tetraborate and 200 mg of sodium dodecyl sulfate. Later, 176 mg of DTT was added, and the solution was adjusted to 200 mL with deionized water (DI). Serine (0.9516 meqv/L) was used as the standard. In total, 400 µL of the sample or standard serine was mixed with 3 mL of the OPA reagent. The mixtures were incubated for 2 min at room temperature, and the absorbance of the formed chromophore was read on a UV/VIS spectrophotometer (UV-1700, Shimadzu, Kyoto, Japan) at a wavelength of 340 nm. The free amino groups from the proteins were expressed in total serine mEq/g protein.

#### 2.4.2. Enzymatic Hydrolysis of the Proteins

The protein concentrates were subjected to the enzymatic hydrolysis process using food-grade Alcalase 2.4L (Novozymes, Denmark) with an enzyme activity of 2.45 ± 0.07 AU/g. The enzyme–substrate ratio was established at 1:100 (*w*/*w*), the reaction temperature was at 50 °C, and the pH was 8.0. The temperature and the pH remained constant throughout the hydrolysis period (15 or 60 min) using a heating bath and adding 4 M NaOH. After the reaction period, the pH was adjusted to 4.5 with a 4 M HCl solution, and the mixture was frozen and lyophilized [21,27]. The hydrolyzates were ground and sieved through a number 60 mesh (250 µm size) and were stored at 4 °C until use. The degree of hydrolysis (DH%) was determined according to Equation (1) [28].

(1) $$DH\left(\%\right)=\frac{N_{i}-N_{T}}{N_{T}-N_{0}}\times 100$$

where:

*N_i_*: total mEq serine/gram protein in the hydrolyzed sample;
*N*_0_: total mEq serine/gram protein in the non-hydrolyzed sample;
*N_T_*: total serine mEq/gram protein in the fully hydrolyzed sample.

#### 2.4.3. N-Acylation of the Proteins

The N-acylation reaction was performed on 10% protein solutions using dodecanoyl chloride (DDC) at a DDC/NH_2_ from a protein molar ratio of 0.5/1. During the 180 min N-acylation reaction, the temperature and pH were kept constant at 50 °C and pH 10.0 by adding a 4 M NaOH solution [21]. Subsequently, the mixture was lyophilized, and the powder was sieved and stored at 4 °C. The amino groups that reacted determined the degree of acylation (DA) as per Equation (2).

(2) $$DA(\%)=\frac{N_{0}-N_{a}}{N_{0}}\times 100$$

where

*N_a_*: total mEq serine/gram protein in the acylated sample;
*N*_0_: total mEq serine/gram protein in the non-acylated sample.

#### 2.4.4. N-Cationization of the Proteins

The N-cationization reaction was performed on 10% protein solutions using glycidyltrimethylammonium chloride (GTMAC) in a GTMAC/NH_2_ molar ratio of the protein used at 4/1. After 60 min of the N-cationization reaction, the temperature and pH were kept constant at 60 °C and pH 10.0 by adding a 4 M NaOH solution [29,30]. Subsequently, the mixture was lyophilized, and the powder was sieved and stored at 4 °C. The degree of cationization (DC) was determined by the amino groups that reacted according to Equation (3).

(3) $$DC\left(\%\right)=\frac{N_{0}-N_{c}}{N_{0}}\times 100$$

where

*N_a_*: total mEq serine/gram protein in the cationized sample;
*N*_0_: total mEq serine/gram protein in the non-cationized sample.

### 2.5. Characterization of Protein Structural Modification

Native and modified proteins were characterized by the secondary structure of proteins, solubility, z-potential, isoelectric point, molecular weight, content of free thiol groups, absorption capacity of water and fat, capacity and emulsifying stability, differential scanning calorimetry, and gelation temperature.

#### 2.5.1. Infrared Transmission Spectroscopy (FT-IR) and Secondary Structure of Proteins

The FT-IR analysis was performed on the protein isolates by dry employing a PerkinElmer II FT-IR spectrometer (Thermo Fisher Scientific, Waltham, MA, USA). The infrared spectra were taken in a range from 4000 to 400 cm^−1^ (0.5 cm^−1^ of resolution) in duplicate for each sample. The spectra obtained were subjected to Fourier auto-deconvolution (FSD) of the overlapping peaks in the amide-I region (1700 to 1600 cm^−1^) using PeakFit^®^ V4.12 software. The relative proportions of the different secondary substructures were determined by calculating areas under the curve of the peaks obtained in the amide-I region [31].

#### 2.5.2. Solubility of the Proteins

The solubility of the proteins as a function of the pH between the range of 2 and 12 was determined. The pH was adjusted with solutions of 4 M NaOH and 4 M HCL. The aqueous dispersions at 1 mg/mL were homogenized for 30 min and centrifuged for 15 min at 14,000× *g*. The obtained supernatant was separated and dried in a convection oven at 105 °C until constant weight was achieved. The percentage of protein solubility was calculated with the difference between the initial and the solubilized weight [27,32].

#### 2.5.3. Isoelectric Point (Ip) and z-Potential

The isoelectric point was determined from the z-potential vs. the pH plot, and the Ip corresponds to the point where the potential becomes 0 mV. The z-potential was measured on 0.1% protein solutions at a pH range from 2 to 10 using a Zetasizer (Nano ZS90 Malvern^®^, Malvern Instruments GmbH, Kassel, Malvern, UK) [33]. pH was adjusted with 0.1 M NaOH and HCl solutions.

#### 2.5.4. Molecular Weight (MW)

MW was determined by gel electrophoresis on a polyacrylamide–sodium dodecyl sulfate plate (12%, SDS-PAGE) using a Mini-Protean^®^ system (Bio-Rad, San Diego, CA, USA) run at 150 V. Proteins were then stained with Coomassie blue R-250. A protein weight marker (11–245 kDa, Biolabs^®^, Torrance, CA, USA) was used as a reference standard [34].

#### 2.5.5. Free Sulfhydryl (SH) Content

Sulfhydryl groups were determined according to Ellman [35]. Briefly, 15 mg of the sample was suspended in 5.0 mL of Tris buffer (0.086 M Tris, 0.09 M glycine, 4 mM EDTA, pH 8.0); 50 μL of the Ellman reagent was added (4 mg DTNB (5.5-dithio-bis-(2-nitrobenzoic acid) to 1 mL of Tris-glycine buffer). Samples were stored for 1 h in darkness at room temperature (25 ± 1 °C), and the absorbances were then read at 412 nm UV-1700 (Shimadzu, Kyoto, Japan). SH content was calculated using the extinction coefficient of 2-nitro-5-thiobenzoate (NTB) (13,600 M^−1^ cm^−1^) and expressed as μmol/g protein.

#### 2.5.6. Water-Holding Capacity (WHC) and Fat Absorption Capability (FAC)

WHC and FAC were determined according to the method described by Boye et al. in 2010 [36]. WHC was assessed by mixing 1 g of the sample and 15 mL of water DI for 30 min. Samples were centrifuged at 2000× *g* for 10 min. The supernatant was removed, and the WHC was expressed as the percentage of water absorbed by the proteins. FAC was determined by mixing ~0.5 g of the sample with 3 mL of sunflower oil, followed by homogenization for 1 min. Subsequently, samples were centrifuged at 4000× *g* for 30 min, and the supernatant was discarded. The resultant residue in the tubes was weighed, and the FAC was expressed as the percentage of oil absorbed by the proteins.

#### 2.5.7. Emulsifying Capacity

The emulsifying capacity was determined by the Boye et al., (2010) methodology [36]. Briefly, 1.5 mL of oil was added to 4.5 mL of a protein solution (0.5% *w*/*v*) prepared in a 0.01 M phosphate buffer (pH 7). The mixture was homogenized at 20,000 rpm at 25 °C for 2 min using a 5 mm dispersing shaft (ds-500/5, Dlab). Subsequently, 250 µL of the emulsion was mixed with 50 mL of 0.1% sodium dodecyl sulfate solution, and the resulting absorbance was read at 500 nm, employing a UV/VIS spectrophotometer (UV-1700, Shimadzu, Kyoto, Japan). The emulsifying activity index (EAI) and emulsifying stability index (ESI) were calculated, as described by Pearce and Kinsella (1978) [37]:

(4) $$EAI\;(m^{2}/g)=\frac{2\;x\times 2.303\;x\times A_{0}\;x\times N}{c\;x\times \varphi \;x\times 10000}$$

(5) $$ESI\;(m^{2}/g)=\frac{A_{0}\;x\times t}{∆A}$$

where *A*_0_ corresponds to the absorbance of the diluted emulsion right after homogenization, *N* is the dilution factor (×150), *c* is the protein height per volume (g/mL), *φ* is the oil volume fraction of the emulsion, Δ*A* is the change in absorbance between 0 and 10 min (A0–A10), and *t* is the time interval of 10 min [36].

Also, the z-potential of the emulsions was measured using a Zetasizer (Nano ZS90 Malvern^®^, UK) as a stabilized parameter.

#### 2.5.8. Differential Scanning Calorimetry (DSC)

The thermal behavior of the proteins was evaluated using a DSC device (Q2000, TA Instruments, New Castle, DE, USA) calibrated with indium at a modulated temperature. Approximately 2.0 mg of the sample was placed in a sealed aluminum crucible under a constant stream of nitrogen (30 mL/min). A vacuum-sealed aluminum crucible was used as a reference. Thermograms between 20 and 150 °C were collected at a heating rate of 10 °C/min [38].

#### 2.5.9. Gelation Temperature (Tgel)

The temperature sweep on a rheometer (MCR92, Anton Paar Corporation, Graz, Austria) using a C-CC27/T200XL concentric cylinder geometry was used to determine protein gelation temperature (Tg). In order to induce the gel formation, samples (5% of protein, *w*/*v*) were submitted to a heating ramp composed of two steps: (i) from 25 °C to 80 °C, followed by (ii) 80 °C for 5 min. The heating and cooling rates were both 1 °C/min. The phase shift angle (δ) and the storage (G′) and loss (G″) modules were measured at a 0.1% shear strain and an angular frequency of 1.0 rad/s, where the protein’s solution remained within the linear viscoelastic region [39].

Also, frequency sweep was determined for the protein solution before and after the temperature sweep. The linear viscoelastic region (LVR) was evaluated at a 1 rad/s constant angular frequency and a shear strain ranging from 0.001% to 100% [40]. Once the LVR was calculated for each type of protein, frequency sweeps were performed by varying the angular frequency from 10 to 0.1 rad/s at a constant temperature of 25 °C. This test measures the storage module (G′) and the phase shift angle (δ) to assess the gel strength of the protein gel [41].

### 2.6. Encapsulating Capacity Assessment

#### 2.6.1. Annatto Seed Extract

The annatto seed extract was obtained by leaching with 80% ethanol at a 1:5 *w*/*v* ratio for 48 h under constant stirring [42]. The extract was concentrated using a rotary evaporator (R-114, BÜCHI^®^) at 60 mbar and 35 °C. The concentrated extract was freeze-dried until use. The Bixin content was determined in the freeze-dried extract, which was used to determine the encapsulation efficiency (EE). Both methodologies are described later.

#### 2.6.2. Encapsulation Process

The encapsulation process used was according to the described method by Quintero et al. in 2020 [15]. The extracts of native and modified proteins from each plant source (10% of protein, *w*/*v*) were suspended in DI water and stirred at 25 °C for 12 h. The dispersions were adjusted to pH 9.0 with 0.2 M NaOH and heated at 80 °C for 1 h. Subsequently, the annatto extract or hydrophilic dye at a 1:5 core/wall ratio was added, using a homogenizer Ultra-Turrax T18 (IKA-Labortechnik) operated at 8000 rpm for 1 min. The homogenized dispersions were manually extruded using a 21-gauge needle syringe on a 10% solution of CaCl_2_. The capsules formed and stood in the solution for 15 min. Afterward, the capsules were immersed in a 0.01% glutaraldehyde solution for 1 min, washed with DI water, and filtered (11 µm of cellulose). The supernatant from each encapsulation process was used to determine the encapsulation efficiency for the protein.

#### 2.6.3. Quantification of Bixin in the Extract and the Supernatant

The previously diluted annatto seed extract (~0.1 g of freeze-dried sample suspended in 30.0 mL of DI water) was diluted with acetone to obtain an absorbance below 1.0 at 486 nm, employing a Genesys 10S UV/Vis spectrophotometer (Genesys 10S UV-Vis, Thermo Scientific, Waltham, MA, USA). The mg of bixin per gram of the sample was calculated from a calibration curve of a bixin standard built between 12.5 mg/L and 0.19 mg/L [10].

#### 2.6.4. Quantification of Hydrophilic Dye in the Supernatant

The dye concentration in the supernatant of the encapsulation process was determined using a dye calibration curve between 15 mg/L and 500 mg/L. Absorbances of the solutions were read at 524 nm on a Genesys 10S UV/Vis spectrophotometer (Genesys 10S UV-Vis, Thermo Scientific, Waltham, MA, USA).

#### 2.6.5. Encapsulation Efficiency

An indirect methodology determined the encapsulation efficiency of annatto extract and hydrophilic dye [43]. The encapsulation efficiency of the extract was calculated using Equation (6):

(6) EE=((ma−mb)/ma)×100

where *ma* is the concentration of the encapsulated bioactive compounds—bixin or hydrophilic dye in the dispersions before encapsulation—and *mb* is the concentration of the bioactive compounds in the supernatant after the encapsulation process.

### 2.7. Statistical Analysis

Results are presented as means of three replicates and standard deviations (SDs) according to the normality of the data. Statgraphics^®^ Centurion XVI software (XVI version, StatPoint Technologies, Inc., Warrenton, VA, USA) performed the analysis. Differences among treatment means were tested using Fisher’s least significant difference (LSD) test (*p-*value ˂ 0.05).

## 3. Results

### 3.1. Protein Structural Modification

The modification degrees of the proteins are shown in Table 1. The results obtained by the modification degree of the proteins through enzymatic hydrolysis, N-acylation, and N-cationization showed that the proteins obtained comparable values in the enzymatic hydrolysis process at 15 and 60 min, regardless of the protein source. However, chemical processes, such as N-acylation and N-cationization, showed a statistically significant difference (*p-*value < 0.05) between the sources.

QP was the plant protein that presented the lowest percentage of modification for N-acylation and N-cationization (10.53% and 7.89%, respectively), followed by SP (42.63% and 34.21%, respectively) and LP (67.18% and 66.64%, respectively).

### 3.2. Characterization of Protein Structural Modification

#### 3.2.1. Effect of the Protein Modifications on Their Secondary Structure

**Figure 1:**
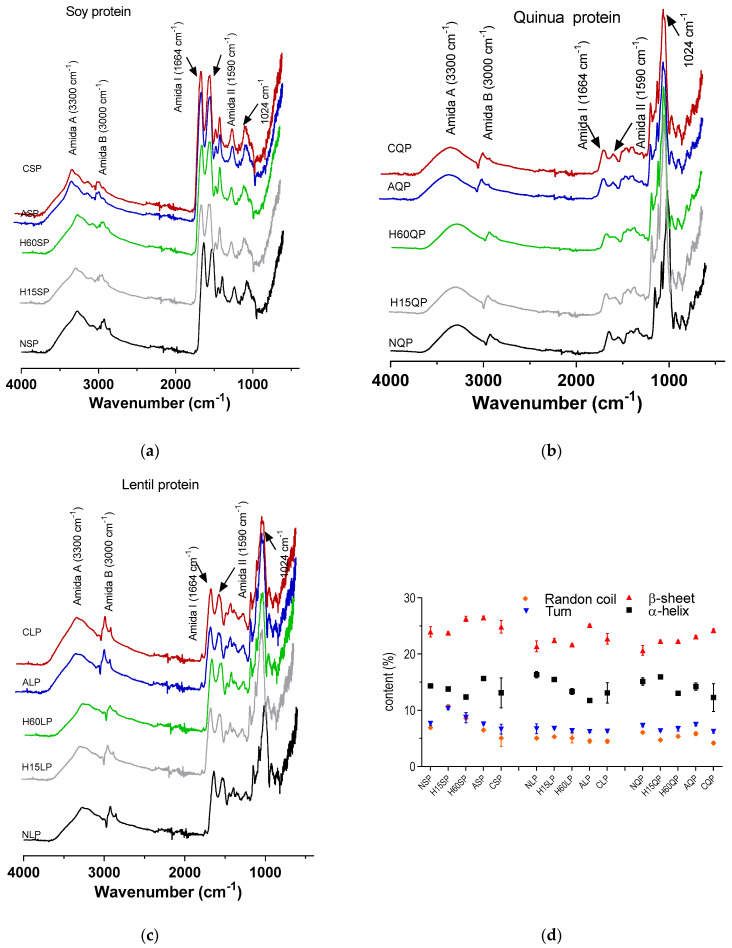
FT−IR spectrum of native and modified (**a**) soy protein, (**b**) quinoa protein, and (**c**) lentil proteins by N-acylation, N-cationization, and enzymatic hydrolysis. (**d**) Content of folds (α helices, β sheets, random coil, and turn) in the secondary structure of native and modified proteins by N-acylation, N-cationization, and enzymatic hydrolysis. NSP: native soy protein, NQP: native quinoa protein, NLP: native lentil protein, ASP: N-acylated soy protein, AQP: N-acylated quinoa protein, ALP: N-acylated lentil protein, CSCP: N-cathinized sodium caseinate, CSP: N-cathinized soy protein, CQP: N-cathinized quinoa protein, CLP: N-cathinized lentil protein, H15SP: hydrolyzed soy protein 15 min, H60SP: hydrolyzed soy protein 60 min, H15QP: hydrolyzed quinoa protein 15 min, H60QP: hydrolyzed quinoa protein 60 min, H15LP: hydrolyzed lentil protein 15 min, and H60LP: hydrolyzed lentil protein 60 min.

Once the proteins were structurally modified and dried, FT-IR spectra were evaluated, and the deconvolution of each spectrum in the amide I region (1700 to 1600 cm^−1^) was analyzed. This region is the most sensitive to estimating the proportion of the different folds in the secondary structures (β sheets, α helix, random coils, and turn). Figure 1 shows the FT-IR spectra for native and modified plant proteins and the percentage of the protein folding of its secondary structure.

The bands corresponding to the emission ranged between 3000 and 3500 cm^−1^, corresponding to the protein structure’s OH-, NH-groups, and some COO groups of amino acids. These bands showed an increase in the number of proteins modified by N-acylation and N-cationization without differences in the protein source. This increase is more significant for LP than QP and SP, which present a higher percentage of the degree of modification (Table 1). The characteristic bands for amide I and amide II were presented for all the samples, regardless of the source and modification; however, the samples modified by enzymatic hydrolysis showed a slight decrease in the definition of these bands [44].

#### 3.2.2. Effect of Protein Modification on Denaturation Temperature

**Figure 2:**
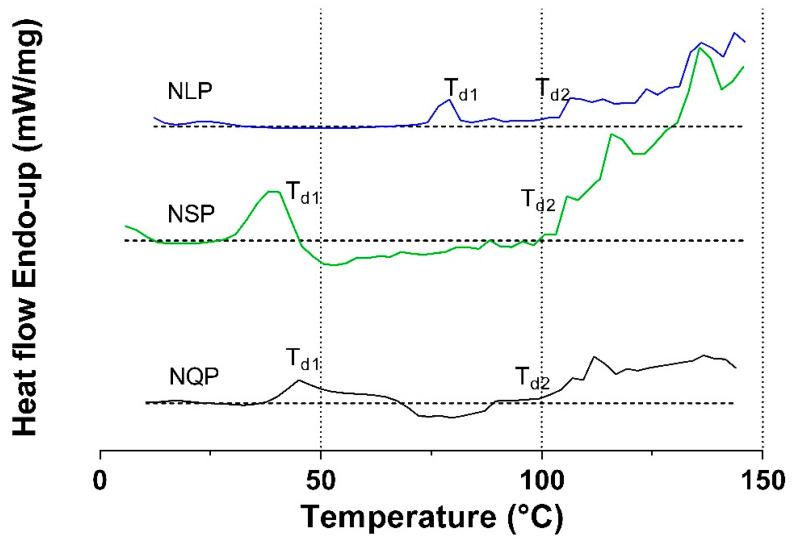
Differential scanning calorimetry (DSC) thermogram of native quinoa, lentil, and soy proteins. NSP: native soy protein, NQP: native quinoa protein, NLP: native lentil protein, Td1: denaturation temperature, and Td2: degradation temperature.

The DSC technique is usually employed to determine most biopolymers’ glass phase transitions or denaturation, including proteins. Figure 2 shows the thermograms of the native SP, QP, and LP powders with humidity between 5% and 10%. In general, protein thermograms report two endothermic peaks. The first (Td1) between 40 °C and 80 °C is associated with the decomposition of weak interactions (hydrogen bonds, hydrophobic and electrostatic interactions), which leads to the denaturation of proteins; the second peak above 100 °C (Td2) is associated with the decomposition of the superimposed protein with the evaporation of water [45].

From DSC, Table 2 reports the Td1 and Td2 obtained for all the proteins studied. In general, proteins from native and modified plant sources did show peaks attributed to Td1, except for hydrolyzed proteins.

#### 3.2.3. Effect of Protein Modification on Their Functional Properties

**Figure 3:**
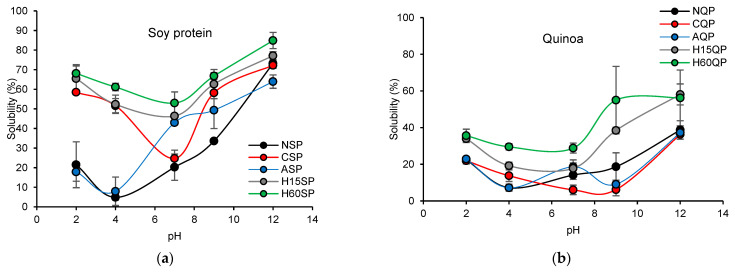

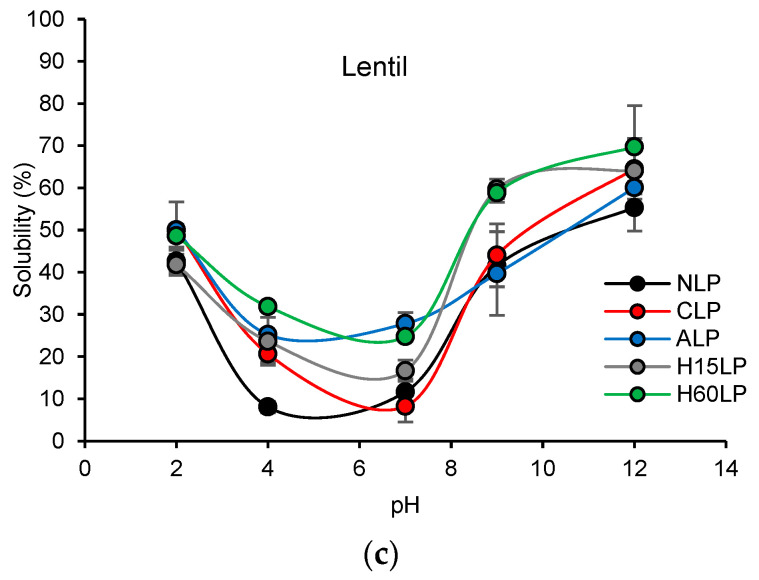
Effect of pH on the solubility profile of native and modified proteins by N-acylation, N-cationization, and enzymatic hydrolysis. (**a**) Soy proteins, (**b**) quinoa proteins, and (**c**) lentil proteins. NSP: native soy protein, NQP: native quinoa protein, NLP: native lentil protein, ASP: N-acylated soy protein, AQP: N-acylated quinoa protein, ALP: N-acylated lentil protein, CSCP: N-cathinized sodium caseinate, CSP: N-cathinized soy protein, CQP: N-cathinized quinoa protein, CLP: N-cathinized lentil protein, H15SP: hydrolyzed soy protein 15 min, H60SP: hydrolyzed soy protein 60 min, H15QP: hydrolyzed quinoa protein 15 min, H60QP: hydrolyzed quinoa protein 60 min, H15LP: hydrolyzed lentil protein 15 min, and H60LP: hydrolyzed lentil protein 60 min.

The solubility profile of proteins as a function of pH was considered a guide for the functionality of native and modified proteins since this is directly related to many functional properties, such as the emulsifying capacity, foam formation, and gel formation [31]. Native soybean, quinoa, and lentil proteins reported a typical U-shaped solubility profile for proteins (Figure 3), where higher percentages of higher solubility were reported at lower pHs, and higher percentages of solubility were obtained by those between pH 4 and 6, close to the pH of the proteins (Table 3, Table 4 and Table 5).

**Figure 4:**
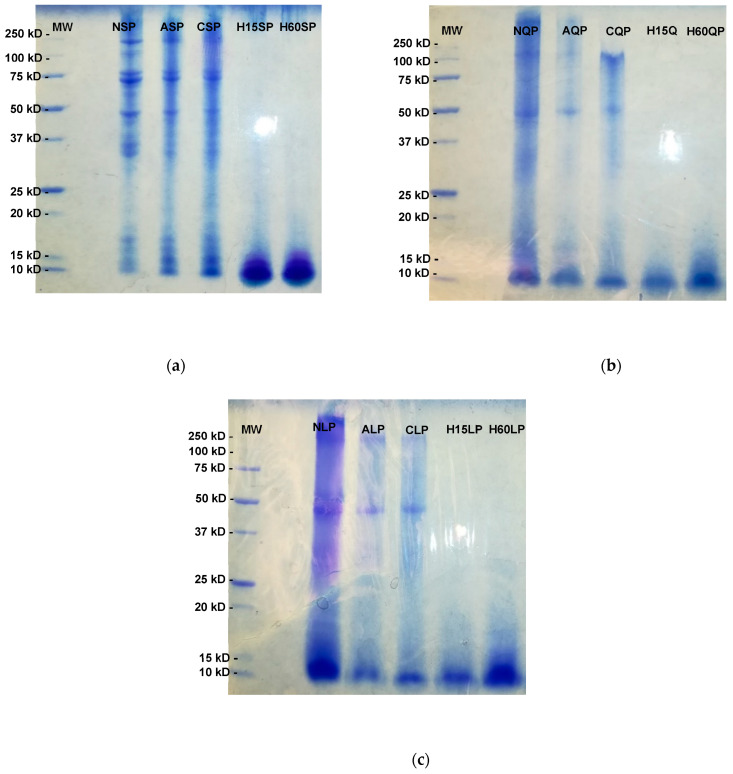
SDS-PAGE of native and modified soybean (**a**), quinoa (**b**), and lentil (**c**) proteins by N-acylation, N-cationization, and enzymatic hydrolysis. NSP: native soy protein, NQP: native quinoa protein, NLP: native lentil protein, ASP: N-acylated soy protein, AQP: N-acylated quinoa protein, ALP: N-acylated lentil protein, CSCP: N-cathinized sodium caseinate, CSP: N-cathinized soy protein, CQP: N-cathinized quinoa protein, CLP: N-cathinized lentil protein, H15SP: hydrolyzed soy protein 15 min, H60SP: hydrolyzed soy protein 60 min, H15QP: hydrolyzed quinoa protein 15 min, H60QP: hydrolyzed quinoa protein 60 min, H15LP: hydrolyzed lentil protein 15 min, and H60LP: hydrolyzed lentil protein 60 min.

The MW of the proteins modified by N-cationization and N-acylation was kept constant compared to the MW of the native proteins. As expected, the MW of the hydrolysis proteins decreased significantly due to the breaking of the peptide bonds on the protein structure, as shown in Table 3, Table 4 and Table 5 and Figure 4.

**Figure 5:**
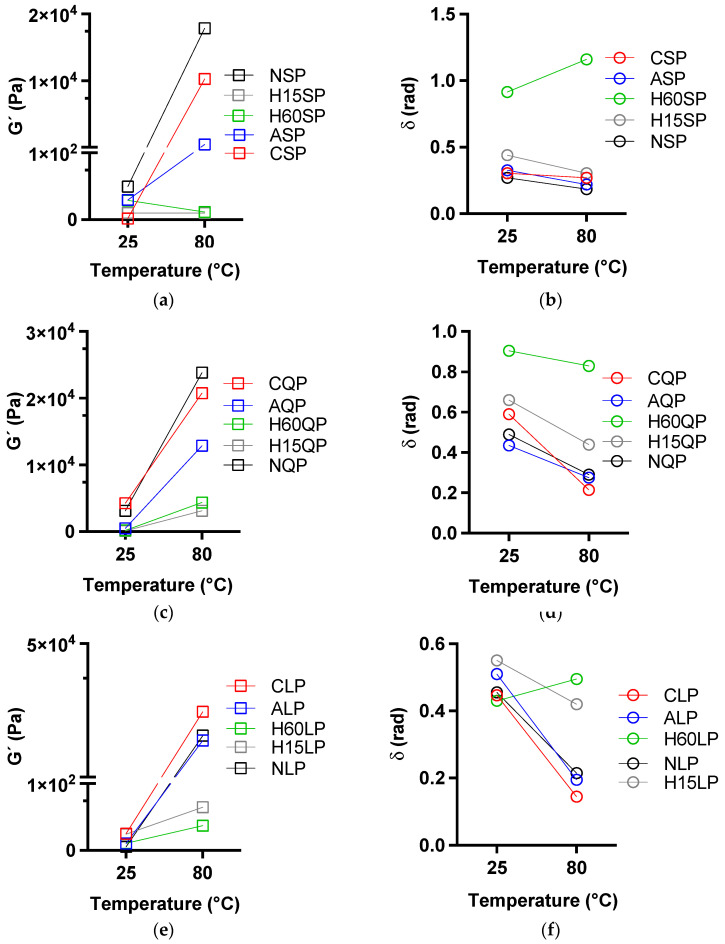
Storage modulus (G′) and phase change angle (δ) at the beginning and the end of the temperature sweep for native- and N-acylation-modified lentils (**a**,**b**), quinoa (**c**,**d**), and proteins (**e**,**f**). N-cationization and enzymatic hydrolysis. NSP: native soy protein, NQP: native quinoa protein, NLP: native lentil protein, ASP: N-acylated soy protein, AQP: N-acylated quinoa protein, ALP: N-acylated lentil protein, CSCP: N-cathinized sodium caseinate, CSP: N-cathinized soy protein, CQP: N-cathinized quinoa protein, CLP: N-cathinized lentil protein, H15SP: hydrolyzed soy protein 15 min, H60SP: hydrolyzed soy protein 60 min, H15QP: hydrolyzed quinoa protein 15 min, H60QP: hydrolyzed quinoa protein 60 min, H15LP: hydrolyzed lentil protein 15 min, and H60LP: hydrolyzed lentil protein 60 min.

**Figure 6:**
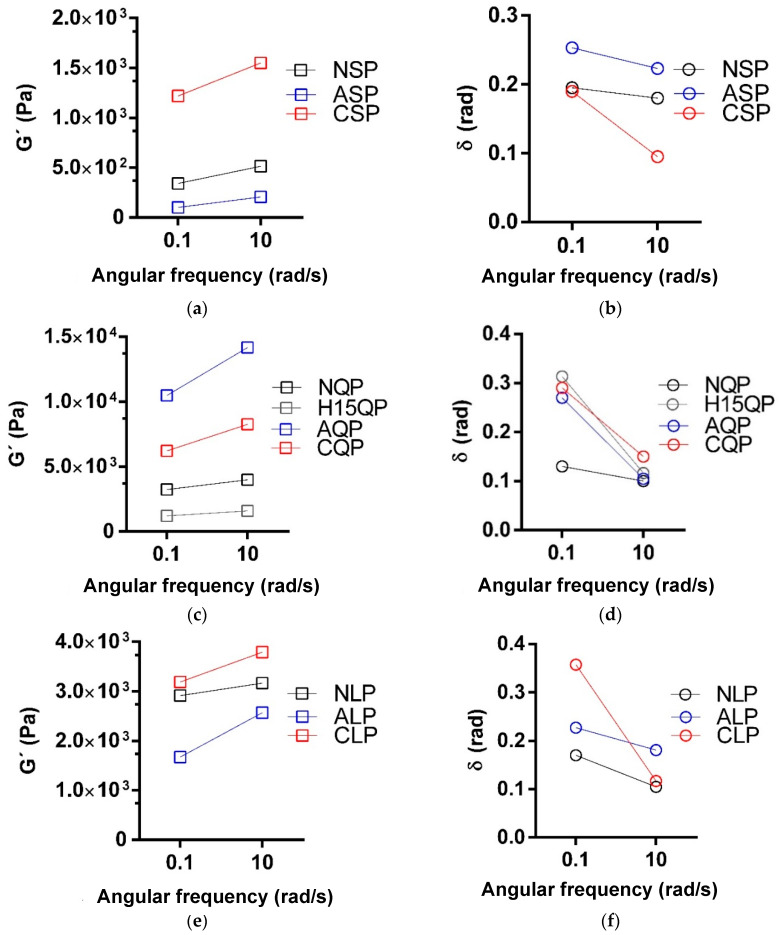
Storage modulus (G′) and phase change angle (δ) at the beginning and end of the frequency sweep for native- and N-acylation-modified lentils (**a**,**b**), quinoa (**c**,**d**), and lentils (**e**,**f**), N-cationization and enzymatic hydrolysis. NSP: native soy protein, NQP: native quinoa protein, NLP: native lentil protein, ASP: N-acylated soy protein, AQP: N-acylated quinoa protein, ALP: N-acylated lentil protein, CSCP: N-cathinized sodium caseinate, CSP: N-cathinized soy protein, CQP: N-cathinized quinoa protein, CLP: N-cathinized lentil protein, H15SP: hydrolyzed soy protein 15 min, H60SP: hydrolyzed soy protein 60 min, H15QP: hydrolyzed quinoa protein 15 min, H60QP: hydrolyzed quinoa protein 60 min, H15LP: hydrolyzed lentil protein 15 min, and H60LP: hydrolyzed lentil protein 60 min.

Figure 5 and Figure 6 relate the G′ and δ in the temperature and frequency sweeps for the formation of protein gels and the mechanical spectrum of the gels formed.

The G′ reported in Figure 5 shows how the elastic fraction—represented by G′—increases significantly with increasing temperatures from 25 °C to 80 °C for protein solutions where a Tg was reported. In the case of hydrolyzed proteins, the G′ was kept either constant or with slight increases in the temperature. The δ tends to decrease with increasing temperature for the solutions that report Tg, reiterating that at lower δ, the gel structure predominates in the system.

Figure 5 shows the mechanical spectra via the frequency sweep test for all systems with gelling capacity after heat treatment.

The behavior of all the gels obtained is the behavior of weak gels, dependent on the frequency, where G′ and δ vary with regard to the applied angular frequency, although the highest G′ is for the proteins modified by N-acylation and N cationization. Regardless of the protein source studied, the resistance of the gels of the native proteins was higher due to the constant values of G′ and δ obtained at angular frequencies of 0.1 and 10 rad/s.

### 3.3. Encapsulation of Annatto Extract and Hydrophobic Dye by Ionic Gelation Using Native and Modified Proteins

Table 6 reports the results of the encapsulation efficiency percentage (EE) of the hydrophilic dye and the annatto extract using the native and modified proteins as coating materials. The maximum EE obtained was for the dye encapsulation process using SP, followed by LP and QP. The encapsulation of the annatto extract showed lower EE compared to the dye encapsulation processes due to the hydrophobic fraction contained in the extract.

Table 7 presents the results of the multifactorial analysis of variance performed to evaluate the effect of protein modification, the bioactive compounds to be encapsulated, and the source of the protein used on the encapsulation efficiency by ionic gelation. The results show the statistically significant effect of the three factors in their linear expression on the EE (with *p*-values < 0.05). At the same time, in the case of interactions on the same response variables, there were no statistically significant effects (*p*-value = 0.053) for the interaction of the protein and active modification to be encapsulated.

**Figure 7:**
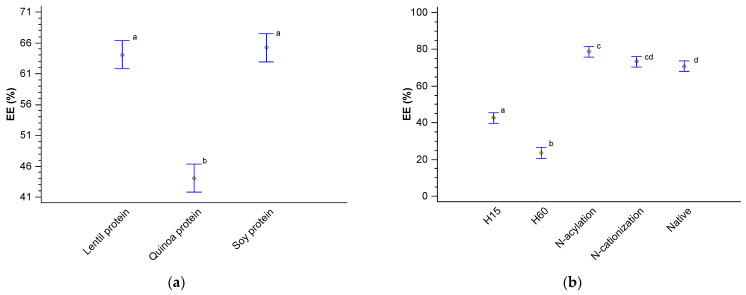

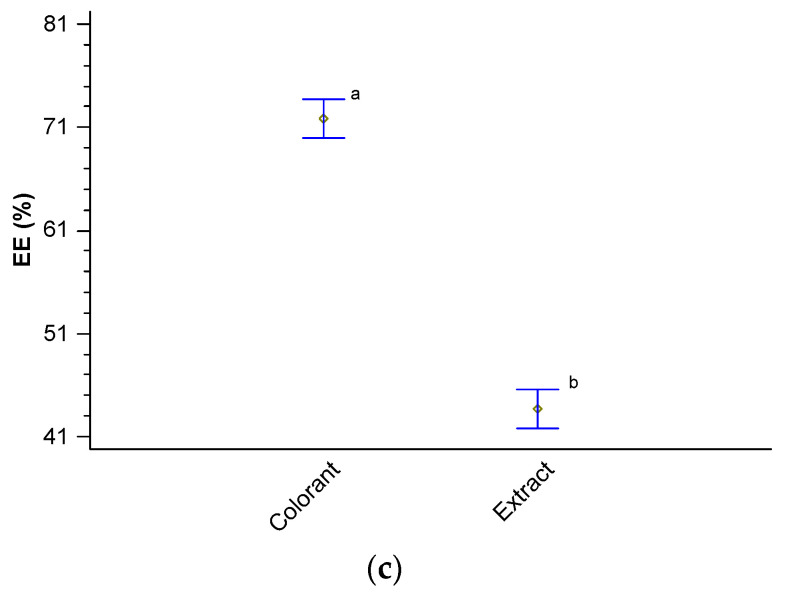
Fisher’s LSD test with 95% confidence for (**a**) encapsulation efficiency (EE) with respect to the protein source, (**b**) EE with respect to the mechanism of derivatization of proteins, and (**c**) EE with respect to the bioactive compounds to be encapsulated.

Figure 7 compares the mean of encapsulation efficiencies (Fisher’s LSD test) using the three different plant proteins, the effect of each modification, and the encapsulation of the dye and annatto extract. Figure 7a shows how LP and SP present a higher EE compared to those obtained with QP, possibly attributed to the difference between MW, a higher SH content, and the higher AW, FAC, and EAI capacities previously reported. Similarly, when comparing the structural modifications of the proteins (Figure 7b), a higher EE was observed for the proteins modified by N-acylation and N cationization than the native proteins, but without statistically significant differences (*p*-value > 0.05). However, the proteins modified by enzymatic hydrolysis presented low EE values with a statistically significant difference (*p*-value < 0.05) compared to the other two modifications and the native proteins. This behavior was attributed to the gel formation capacity of the obtained protein fractions discussed in the previous section. Finally, the difference in EE between the encapsulated bioactive compounds—hydrophilic dye and the annatto extract—a higher EE (*p*-value < 0.05) is clearly seen in Figure 7c for the dye than for the annatto extract in the encapsulation process using the same coating materials.

## 4. Discussion

Results obtained by the modification degree of the proteins by enzymatic hydrolysis, N-acylation, and N-cationization, reported in Table 1, show that the variation in the percentage of protein modification is attributed to the disposition of NH_2_ groups in the protein structure, according to its folding and the steric hindrance that nearby groups can generate in these experimental conditions [18]. For SP isolated by acid precipitation, structural modifications such as N-acylation and N-cationization were evaluated, reporting higher degrees of modification than those obtained in this study—~60% for N-acylation and ~ 92% for N-cationization [18,29]. This shows that the method of obtaining the protein and the protein source influence these two chemical structural modifications. On the other hand, enzymatic hydrolysis using Alcalase 2.4L achieved percentages of plant-based proteins comparable with results reported in the literature [29,46].

Analysis of the secondary structure of native and modified proteins is shown in Figure 1d. The structures of α helices, β leaves, and random coil were observed in the deconvolved spectra according to four main Gauss bands centered at ∼1654, ∼1638, and ∼1670 cm^−1^ [31]. The results obtained reported an average content between ∼5 and 10% of random coils and turns in the secondary structure of the proteins regardless of the source. Furthermore, these random coils and turn percentages held constant for chemical and enzymatic modifications for all proteins (Figure 1d). The α-helix content is usually less constant than β-sheets’ content for native and modified proteins; however, it presents a slight decrease in the enzymatic modifications. The longer the hydrolysis time, the greater the decrease in the α-helix content in the secondary structure of the protein.

This effect was due to the breaking of the peptide bonds caused by the Alcalase 24L; when breaking these bonds, the entire secondary structure is modified. The effects of enzymatic hydrolysis on native proteins are not so marked in the analyses of the FT-IR spectra and secondary structure. The analysis is carried out on a set of proteins obtained from a specific source, with the bands being the weighted expression of all the proteins present. Figure 1d shows that the mean proportion of β-sheets for all proteins, both native and modified, was between 20% and 30%. The results obtained for β-sheets were consistent with those reported for proteins from different plant sources, where β-sheet-like folds are predominant [31]. Xuan Li et al., (2018) reported a percentage of ~29% β-sheets for isolated quinoa proteins [46]. Similarly, Meng and Ma (2001) reported that 36% and 19% of the folding of the secondary structure of bean globulins were β-sheets and α-helices, respectively [47]. On the other hand, it was reported that lentil proteins had ~47% β-sheet structures against ~25% α-helices that were quantified in samples of native lentil proteins [48].

The differential scanning calorimetry (DSC) thermogram of plant proteins reported that the highest Td1s were those reported for LP, regardless of whether it was in its native or modified state, which is due to the close relationship between Td1 with the amino acid composition and the secondary structure of the proteins α-helices, β-sheets, random coils, and turns. The higher the amino acid chain and folding in the protein structure, the greater the energy requirement for denaturation and, therefore, the higher Td1 [31]. This behavior is supported by the results reported in Figure 1 and Figure 3, where it is reported that among the studied protein sources, all presented a comparable percentage in the content of α-helices and β-sheets. Still, LP reported a higher molecular weight than QP and SP. Likewise, Carbonaro et al. (2008) studied the thermal stability of proteins extracted from lentils and black beans, reporting that lentil proteins showed greater thermal stability than bean proteins, mainly attributed to the higher content of β-sheets [49]. The effect of the hydrolyzed proteins was due to the partial denaturation of the proteins by the enzymes used in the hydrolysis process, which cut the peptide bonds and decreased the molecular weight and folding of the proteins, entailing a lower expenditure of energy on the denaturation processes and, therefore, no endothermic peak. This is in line with Molina and Cañon’s description in their 2001 study of the thermal behavior of soy proteins with and without various degrees of hydrolysis [50]; they determined that increasing the degree of hydrolysis, the endothermic peak, which describes the denaturation process, decreases significantly [50].

Regarding the proteins derivatized by N-acylation and N-cationization, increases in the reported Td1 were obtained (Table 2). Modifications by N-acylation and N-cationization affect protein conformation by promoting the deployment of the secondary structure, which favors functional properties, such as protein solubility [29]. By increasing the solubility of the protein, the hydrogen bonds with water increase, and the proteins have more bound water than native proteins; therefore, a higher temperature is necessary for protein denaturation [51].

In general, the percentages of solubility increased at neutral and low pHs when executing the hydrolysis process of proteins compared to native proteins. In doing so, soluble peptides and more exposed ionizable carboxyl and amino groups are obtained on the protein, improving solubility [31]. Due to the degree of hydrolysis being so close between the 15 min and 60 min treatments for all protein sources (Table 1), the solubility profiles are very comparable between the same protein source for both hydrolysis times. These results coincide with other reports where the hydrolysis process favors the solubility of soy, lentil, and quinoa proteins [52,53,54].

The solubility profiles of the proteins modified by N-cationization and N-acylation presented variations compared to the profiles of native proteins proportional to the percentage of the degree of modifications reported in Table 1. The proteins modified by N-cationization obtained a profile with higher solubility in the acid pH ranges than native proteins. However, these same proteins reported lower percentages of solubility close to pH 7. The cationization increased the net number of positive charges for the protein and reduced the number of NH_2_ available. This structure change modified the general balance between acidic and basic groups in the native protein, resulting in a change in the solubility profile, as shown in Figure 3. Likewise, the proteins derivatized by N-acylation presented modifications in their solubility profiles, increasing the percentages of solubility between pH 4 and 9 due to the decrease in reactive NH_2_ groups that had already reacted during the modification processes.

The functional properties of the native and modified proteins are shown in Table 3, Table 4 and Table 5. The type of structural modification was the independent variable that affected the studied parameters. The structural parameters, such as the z-potential (ζ), the isoelectric point (pI) of the proteins, the molecule weight (MW), and the free thiol groups (SHs), presented statistically significant differences (*p*-value < 0.05) between the modified and native protein. ζ values of the native and modified protein dispersions at pH 10—the pH in which the proteins presented their highest percentage of solubility (Figure 3)—reported a decrease for proteins modified by N-cationization and N-acylation compared to native proteins, contrary to hydrolyzed proteins. ζ values obtained for the proteins at pH 10 were negative due to the neutralization of the acid groups and the ionization of the NH_2_ groups on the protein structure by the alkaline medium. The decrease in ζ after the modification processes by N-cationization and N-acylation was due to the modification of the primary amino groups that were replaced by positively charged groups from DDC and GTMAC [30]. The proteins modified by N-cationization and N-acylation, due to having fewer amino groups to neutralize, experienced a decrease in ζ for the native proteins; therefore, the quantification of ζ confirmed the modification of the proteins, which is a relationship that is supported by the percentage of the degree of modification for each protein source reported in Table 1. Concerning the variation in the solubility profile and the ζ of the native and modified proteins, the pI of the proteins presented an increasing trend for the modified proteins by N-cationization and N-acylation; however, they did not show statistically significant differences (*p-*value > 0.05).

The breaking of these peptide bonds in the enzymatic hydrolysis process generated the exposure of the SH groups of the protein that were not free in the native structure. A statistically significant increase in this parameter was observed in the hydrolyzed proteins compared with native proteins. The modified proteins did not show a uniform behavior of increasing or decreasing SH groups; however, this variation was due to the structural modification generated by the incorporation of DDC and GTMAC, which favors the detachment of the protein tertiary structure, exposing some functional groups that were buried inside the structure in its native state [30].

The chemical and enzymatic modifications studied reported the ability to improve the functional properties of proteins, such as solubility, emulsion, and foam formation capabilities, increased hydrophobicity, and swelling power, which are results that coincide with those obtained in this study [18,55]. Functional properties such as WHC, FAC, and EAI; the stability of the emulsions determined by ESI and the Z potential in the emulsion (ζe); and the gel formation temperature (Tgel) of the native and modified proteins are shown in Table 3, Table 4 and Table 5. WHC and FAC were functional properties that presented variations due to the structural modifications of the proteins regardless of the source studied. WHC in the majority of cases did not present statistically significant differences (*p-*value > 0.05) between the modified proteins and the native protein. However, the enzymatic hydrolysis processes did show a statistically significant decrease (*p*-value ˂ 0.05) for WHC, and the N-cationization processes showed an increasing trend for WHC despite not showing statistically significant differences (*p*-value > 0.05).

On the other hand, FAC reported an increase with statistically significant differences (*p*-value ˂ 0.05) for proteins modified by both chemical and enzymatic processes. In the case of enzymatic hydrolysis, the decrease in WHC was probably attributed to the reduction in the length of the protein’s molecular chain, which generated the greater exposure of the hydrophobic regions of the protein that decreased WHC and, in turn, increased FAC [17,29]. Regarding N-cationization, the incorporation of the polar groups on the protein structure increased its amphiphilic character, which, in turn, affected the protein conformation by promoting the deployment of the tertiary structure, further exposing the embedded hydrophobic groups in the structure, favoring the WHC and FAC [17,29]. Finally, the binding of the fatty acid chains to the protein at the N-acylation process also increased the amphiphilic properties of the protein, which explains the increase in FAC in all the proteins modified by this chemical process [17,29].

The EAI, ESI, and ζe obtained for the proteins in their native and modified states presented statistically significant differences (*p-*value ˂ 0.05). ζe is considered as a factor related to the stability of the emulsions since at absolute values of surface charge (ζ) greater than ±30 mV, the repulsion of the oil droplets in the colloidal system was generated and, therefore, had better stability against coalescence [56]. This behavior of colloidal systems stabilization was related to the solubility changes, the WHC, and the FAC of the proteins attributed to the structural changes, such as the reduction in the polypeptide chain and the incorporation of the hydrophobic and polar groups explained above. In the case of soy proteins (Table 3), the EAI did not present statistically significant differences between the modified proteins and the native protein (*p*-value > 0.05); however, the ESI and ζe presented an increase with a statistically significant difference (*p*-value ˂ 0.05) for ASP, CSP, and H15SP compared to native proteins (NSPs). H60SP exhibited a decrease in the parameters related to the stability of the emulsion, possibly due to the increase in the degree of hydrolysis, which, by further breaking the polypeptide chains of the protein, generated short chains of hydrolyzed proteins that could not efficiently wrap the drop of oil, and therefore, were less stable. QP showed a comparable behavior for EAI with soy proteins (Table 4), differentiating the significant increase in EAI for H15QP compared with NQP and a decrease for H60QP compared with NQP. In this case, enzymatic hydrolysis generated a greater effect than the chemical modifications used, possibly due to the difference in the low percentages of the degree of modification (Table 1) obtained for QP between the different modifications. The stability of the emulsions obtained with the native and modified quinoa proteins showed comparable behavior with the SP described above. However, the variation is proportional to the degree of modification. Finally, the lentil proteins presented a lower EAI (Table 5) but better emulsion stability—ESI and ζe—than the proteins from the other two sources studied. Despite these differences, the trend in the behavior of the parameters of EAI, ESI, and ζe was the same as those previously described based on the types of modifications studied.

The reported gelation temperature (Tg) for the native and modified proteins is shown in Table 3, Table 4 and Table 5. The temperature sweep used for the Tg determination uses the storage modulus (G′) and the phase change angle (δ) as parameters to define the temperature where the change in protein dispersion to a gel-like system is promoted due to the formation of disulfide bonds and electrostatic interactions [57,58]. Furthermore, δ determines the hardness of the gel obtained. The Tg of all the proteins modified by N-cationization and N-acylation increased regardless of the source of the protein, while for the hydrolyzed proteins, there was no gel formation (Table 3, Table 4 and Table 5). The increase in Tg of the proteins modified by N-cationization and N-acylation coincides with the results obtained in the thermograms reported in Table 2, specifically in Td1, which refers to the denaturation temperature of the proteins. The impossibility of the fractions of the hydrolyzed protein molecules to form an adequate network structure during the heat treatment does not allow the hydrolyzed proteins to form the gel. This behavior has been previously reported for proteins from other plant sources [27].

Microencapsulation processes by ionic gelation are commonly performed using sodium alginate as the coating material. Alginate refers to a group of naturally occurring anionic polysaccharides extracted from brown algae. These linear polymers consist of 1,4-linked β-D-mannuronic acid (M) and 1,4 α-L-guluronic acid (G) chains, which can be arranged in either homogenous (poly-G, poly-M) or heterogenous (MG) block-like patterns. In this process, the COO- groups of alginates react with calcium ions present in the gel-forming solution, resulting in the formation of a hydrogel that encapsulates bioactive compounds of interest.

The encapsulation efficiency in ionic gelation processes is attributed to the material’s ability to form gels in the presence of ions through directed covalent bonds. Additionally, the affinity between the coating material and the active compound before gel formation plays a crucial role. Greater affinity and solubility between the material, the active compound, and the medium can lead to higher encapsulation efficiency [59]. These parameters explain how protein structural modifications, protein sources, and the bioactive compound’s linear expression interact statistically to affect encapsulation efficiency, as observed in Table 7 and Figure 7. For instance, modifying protein structures through hydrolysis can expose negatively charged radical groups like COO-, which are abundant in aspartic and glutamic amino acids found in plant proteins. Increasing exposure to these compounds enhances gelation efficiency but may also impact encapsulation efficiency. Incorporating basic and acidic groups into protein structures similarly modifies charges and gel formation processes, potentially decreasing efficiency.

Some of the results obtained agree with those reported by other authors. Nesterenko et al., between 2013 and 2014, evaluated the structural modification of soybean and sunflower seed proteins as a strategy to increase the encapsulation efficiency of α-tocopherol by spray drying [7,17,21,29,30]. They applied enzymatic hydrolysis, N-acylation, and N-cationization to increase both proteins’ functional properties and encapsulation efficiency. N-acylation was the modification that achieved the highest increase for both proteins, proceeding from a native state encapsulation efficiency of 79.7% and 92.6% for soy and sunflower seeds proteins, respectively, to 94.8% and 99.6% for the same proteins modified by N-acylation.

If different behaviors comparable to those reported by Nesterenko et al. were obtained regarding the modifications of the proteins’ functional properties, the encapsulation efficiencies would be much lower than those obtained in Nesterenko et al.’s study, and the variations in the modifications did not show any differences. Statistical significance obtained in our study did occur in the one reported by Nesterenko and company. This is mainly attributed to the encapsulation methods employed. While Nesterenko et al. employed spray drying, we standardized and employed the ion gelation encapsulation process. On the other hand, comparing the EE obtained in the encapsulation processes of natural extracts by ionic gelation using sodium alginate as a coating material, we found EE reports to be comparable to those obtained with proteins in their native state and modified by N-acylation and N-cationization. Belščak-Cvitanović et al., 2018, encapsulated cocoa husks, poppy, and hemp bioactive compounds, reporting 73% EE for calcium alginate hydrogel particles obtained by external ionic gelation [59]. Likewise, the results of Moura et al. in 2018 [60] obtained the EE of polyphenols from the extract of *Sambucus nigra* L. for 74.4% and 88.5% of particles obtained by external ionic gelation.

## 5. Conclusions

The structural modification of quinoa, lentil, and soy proteins by enzymatic hydrolysis, N acylation, and N-cationization favored the functional properties of proteins, such as their solubility, water and oil absorption capacity, emulsion capacity, and stability. However, enzymatic hydrolysis did not favor the formation of gels or the ability to encapsulate them by the ionic gelation method. The structural modifications generated on the proteins by N-acylation and N-cationization increased but did not generate statistically significant differences (*p*-value > 0.05) in the encapsulation efficiency of both water-soluble compounds (hydrophilic dye) and the extract of annatto compared to those obtained in native proteins.

## Figures and Tables

**Table 1:** Modification degree of native proteins.

Source	Modification	Modification Degrees (%)
ASP	N-acylation whit DDC	42.63 ± 0.43 ^b^
AQP		10.53 ± 0.55 ^c^
ALP		67.18 ± 0.30 ^a^
CSP	N-cationization whit GTMAC	34.21 ± 0.56 ^b^
CQP		7.890 ± 0.95 ^a^
CLP		66.64 ± 0.18 ^c^
H15SP	Enzymatic hydrolysis	16.46 ± 0.33 ^b^
H15QP		21.04 ± 0.74 ^c^
H15LP		22.73 ± 0.22 ^c^
H60SP		19.86 ± 0.17 ^b^
H60QP		19.79 ± 0.44 ^a^
H60LP		22.93 ± 0.18 ^c^

Different superscript letters (a–c) within modification indicate significant differences between samples (*p*-value ˂ 0.05) according to Fisher’s least significant difference (LSD Fisher) test; values are expressed as mean ± standard deviation (SD) (n = 3). ASP: N-acylated soy protein, AQP: N-acylated quinoa protein, ALP: N-acylated lentil protein, CSP: N-catinized soy protein, CQP: N-catinized quinoa protein, CLP: lentil protein N-catinized, H15SP: hydrolyzed soy protein 15 min, H60SP: hydrolyzed soy protein 60 min, H15QP: hydrolyzed quinoa protein 15 min, H60QP: hydrolyzed quinoa protein 60 min, H15LP: hydrolyzed lentil protein 15 min, and H60LP: hydrolyzed lentil protein 60 min.

**Table 2:** Evaporation and denaturation temperatures of soy, quinoa, and lentil proteins, both native and modified, by N-acylation, N-cationization, and enzymatic hydrolysis.

Source	Td_1_ (°C)	Td_2_ (°C)
NSP	40.69 ± 0.35	105.7 ± 0.22
H15SP	47.71 ± 1.35	105.07 ± 1.45
H60SP	-----	126.71 ±0.36
ASP	63.67 ± 0.61	106.17 ±0.14
CSP	53.97 ± 0.81	103.97 ± 0.24
NQP	45.11 ± 1.35	97.12 ± 2.14
H15QP	-----	93.24 ± 1.09
H60QP	-----	75.58 ± 1.44
AQP	52.13 ± 2.07	97.11 ± 2.09
CQP	40.97 ± 1.85	103.97 ± 1.99
NLP	74.81 ± 1.37	109.82 ± 1.22
H15LP	-----	104.98 ± 2.47
H60LP	-----	104.99 ± 2.00
ALP	76.74 ± 1.05	104.24 ± 2.47
CLP	70.98 ± 2.07	118.04 ± 1.91

**Table 3:** Properties of native and modified soy proteins by N-acylation, N-cationization, and enzymatic hydrolysis.

Parameter	NSP	ASP	CSP	H15SP	H60SP
ζ pH10 (mV)	−25.54 ± 3.70 ^a^	−20.27 ± 1.05 ^b^	−24.3 ± 2.22 ^a^	−29.70 ± 0.79 ^c^	−31.07 ± 1.26 ^c^
MW (kDa)	250.100 to 10	250.75 to 10	250.75 to 10	≤10	≤10
pI	3.00 ± 1.00 ^a^	3.00 ± 1.00 ^a^	5.00 ± 1.00 ^bc^	4.00 ± 1.00 ^ac^	4.00 ± 1.00 ^ac^
SH (µM SH/g)	1.28 ± 0.08 ^a^	1.60 ± 0.20 ^a^	1.50 ± 0.17 ^a^	3.35 ± 0.16 ^b^	5.28 ± 0.79 ^b^
WHC (%)	621.84 ± 7.27 ^a^	630.13 ± 62.27 ^a^	788.68 ± 48.39 ^b^	201.26 ± 12.79 ^c^	232.14 ± 6.22 ^c^
FAC (%)	328.77 ± 8.30 ^a^	362.80 ± 3.95 ^b^	348.06 ± 2.18 ^a^	312.27 ± 6.31 ^a^	321.47 ± 11.22 ^a^
EAI (m^2^/g)	49.04 ± 0.34 ^a^	42.42 ± 0.19 ^b^	37.97 ± 0.42 ^c^	37.81 ± 0.48 ^d^	33.12 ± 0.06 ^e^
ESI (min)	70.68 ± 1.48 ^a^	73.35 ± 3.79 ^b^	78.12 ± 4.70 ^c^	100.64 ± 6.23 ^d^	67.14 ± 3.73 ^a^
ζ_e_ (mV)	−30.67 ± 4.25 ^a^	−65.33 ± 10.08 ^b^	−65.53 ± 15.35 ^b^	−63.16 ± 1.35 ^b^	−45.66 ± 0.45 ^a^
Tgel (°C)	74.76 ± 1.27 ^a^	79.17 ± 2.7 ^b^	67.01 ± 1.62 ^c^	--------	--------

Different superscript letters (a–e) within modification indicate significant differences between samples (*p*-value ˂ 0.05) according to Fisher’s least significant difference (LSD Fisher) test; values are expressed as mean ± standard deviation (SD) (n = 3). NSP: native soy protein, ASP: N-acylated soy protein, CSP: N-cathinized soy protein, H15SP: hydrolyzed soy protein 15 min, H60SP: hydrolyzed soy protein 60 min, MW: molecular weight, IP: isoelectric point, SH: thiol groups, WHC: water retention capacity, FAC: fat absorption capacity, EAI: emulsifying activity index, ESI: emulsifying stability index, Tgel: gelling temperature, ζ pH10: z potential of the solution at pH 10, and ζe: z potential of the emulsion.

**Table 4:** Properties of native and modified quinoa proteins by N-acylation, N-cationization, and enzymatic hydrolysis.

Parameter	NQP	AQP	CQP	H15QP	H60QP
ζ pH10 (mV)	−32.13 ± 1.64 ^a^	−31.67 ± 3.35 ^a^	−31.31 ± 1.77 ^a^	−24.46 ± 1.21 ^b^	−23.52 ± 1.21 ^b^
MW (kDa)	100 to 50	100 to 50	100 to 50	≤10	≤10
pI	5.00 ± 1.00 ^a^	5.00 ± 1.00 ^a^	6.00 ± 1.00 ^a^	5.00 ± 1.00 ^a^	5.00 ± 1.00 ^a^
SH (µM SH/g)	10.25 ± 0.83 ^a^	9.39 ± 2.03 ^a^	16.43 ± 1.59 ^ab^	10.20 ± 8.95 ^b^	17.09 ± 0.68 ^b^
WHC (%)	106.94 ± 0.24 ^a^	114.32 ± 11.88 ^a^	111.53 ± 8.62 ^a^	71.51 ± 1.84 ^b^	82.35 ± 12.58 ^c^
FAC (%)	174.46 ± 0.23 ^ab^	200.46 ± 9.62 ^a^	190.42 ± 6.43 ^c^	163.26 ± 19.66 ^cd^	174.46 ± 0.24 ^bd^
EAI (m^2^/g)	55.67 ± 0.31 ^a^	57.86 ± 0.11 ^b^	54.09 ± 0.45 ^c^	61.13 ± 0.56 ^d^	50.97 ± 0.048 ^e^
ESI (min)	65.88 ± 3.56 ^ab^	95.89 ± 10.22 ^c^	76.78 ± 4.49 ^b^	76.15 ± 3.30 ^b^	58.38 ± 11.78 ^a^
ζ_e_ (mV)	−32.96 ± 0.75 ^b^	−36.36 ± 0.66 ^ac^	−34.15 ± 2.55 ^ab^	−37.27 ± 1.13 ^c^	−34.06 ± 0.68 ^ab^
Tgel (°C)	68.29 ± 2.98 ^a^	69.15 ± 1.41 ^a^	76.04 ± 4.24 ^b^	--------	--------

Different superscript letters (a–e) within modification indicate significant differences between samples (*p*-value ˂ 0.05) according to Fisher’s least significant difference (LSD Fisher) test; values are expressed as mean ± standard deviation (SD) (n = 3). NQP: native quinoa protein, AQP: N-acylated quinoa protein, CQP: N-cathinized quinoa protein, H15QP: hydrolyzed quinoa protein 15 min, H60QP: hydrolyzed quinoa protein 60 min, MW: molecular weight, IP: isoelectric point, SH: thiol groups, WHC: water retention capacity, FAC: fat absorption capacity, EAI: emulsifying activity index, ESI: emulsifying stability index, Tgel: gelling temperature, ζ pH10: z potential of the solution at pH 10, and ζe:: z potential of the emulsion.

**Table 5:** Properties of native and modified lentil proteins by N-acylation, N-cationization, and enzymatic hydrolysis.

Parameter	NLP	ALP	CLP	H15LP	H60LP
ζ pH10 (mV)	−22.87 ± 1.88 ^a^	−17.27 ± 1.27 ^b^	−14.45± 0.67 ^b^	−35.77± 2.31 ^c^	−31.43 ± 2.58 ^d^
MW (kDa)	250 to 50	250 to 50	250 to 50	≤10	≤10
pI	5.00 ± 1.00 ^a^	6.00 ± 1.00 ^a^	6.00 ± 1.00 ^a^	5.00 ± 1.00 ^a^	5.00 ± 1.00 ^a^
SH (µM SH/g)	15.17 ± 0.60 ^a^	15.48 ± 6.70 ^a^	14.82 ± 1.73 ^a^	14.66 ± 1.09 ^a^	16.41 ± 2.11 ^a^
WHC (%)	171.68 ± 18.37 ^a^	148.44 ± 7.04 ^a^	189.32 ± 10.79 ^a^	72.01 ± 4.32 ^b^	58.49 ± 11.76 ^b^
FAC (%)	214.52 ± 17.93 ^a^	259.93 ± 7.23 ^cd^	265.56 ± 3.08 ^d^	227.80 ± 6.88 ^ab^	243.71 ± 7.35 ^bc^
EAI (m^2^/g)	35.94 ± 0.22 ^a^	39.85 ± 0.08 ^b^	37.81 ± 1.30 ^b^	36.89 ± 0.51 ^c^	33.28 ± 0.05 ^d^
ESI (min)	88.71 ± 1.57 ^a^	130.24 ± 5.80 ^b^	60.09 ± 8.25 ^c^	240.04 ± 24.49 ^d^	145.31 ± 3.31 ^b^
ζ_e_ (mV)	−40.46 ± 4.46 ^a^	−40.33 ± 3.50 ^a^	−32.47 ± 0.96 ^b^	−70.65 ± 4.57^c^	−51.97 ± 2.21 ^d^
Tgel (°C)	64.08 ± 3.07 ^a^	68.29 ± 3.50 ^ab^	70.17 ± 2.98 ^b^	--------	--------

Different superscript letters (a–d) within modification indicate significant differences between samples (*p*-value ˂ 0.05) according to Fisher’s least significant difference (LSD Fisher) test; values are expressed as mean ± standard deviation (SD) (n = 3). NLP: native lentil protein, ALP: N-acylated lentil protein, CLP: N-cathinized lentil protein, H15LP: hydrolyzed lentil protein 15 min, H60LP: hydrolyzed lentil protein 60 min, MW: molecular weight, IP: isoelectric point, SH: thiol groups, WHC: water retention capacity, FAC: fat absorption capacity, EAI: emulsifying activity index, ESI: emulsifying stability index, Tgel: gelling temperature, ζ pH10: z potential of the solution at pH 10, and ζe:: z potential of the emulsion.

**Table 6:** Encapsulation efficiency with native and modified soy, quinoa, and lentil proteins by N-acylation, N-cationization, and enzymatic hydrolysis for annatto extract and hydrophilic dye.

Source	Hydrophilic Dye	Annatto Extract
	EE (%)	EE (%)
NSP	96.32 ± 0.11	64.93 ± 0.86
H15SP	88.03 ± 0.17	27.49 ± 3.40
H60SP	39.08 ± 6.11	14.06 ± 8.17
ASP	96.23 ± 0.09	68.91 ± 0.23
CSP	91.81 ± 2.52	65.33 ± 0.96
NQP	73.63 ± 2.04	45.77 ± 1.95
H15QP	21.43 ± 2.05	19.87 ± 0.77
H60QP	3.02 ± 3.29	5.12 ± 0.98
AQP	88.00 ± 1.47	55.32 ± 6.68
CQP	78.81 ± 0.02	49.24 ± 3.98
NLP	80.52 ± 3.82	63.82 ±2.17
H15LP	78.21 ± 0.18	20.72 ±1.50
H60LP	65.58 ± 1.32	13.88 ± 1.89
ALP	89.29 ± 0.02	74.26 ± 2.45
CLP	87.60 ±0.43	66.85 ± 1.86

Values are expressed as mean ± standard deviation (n = 3). EE: encapsulation efficiency, NSP: native soy protein, NQP: native quinoa protein, NLP: native lentil protein, ASCP: N-acylated sodium caseinate, ASP: N-acylated soy protein, AQP: quinoa protein N-acylated, ALP: N-acylated lentil protein, CSP: N-cathinized soy protein, CQP: N-cathinized quinoa protein, CLP: N-cathinized lentil protein, H15SP: 15 min hydrolyzed soy protein, H60SP: 60 min hydrolyzed soy protein, H15QP: 15 min hydrolyzed quinoa protein, H60QP: 60 min hydrolyzed quinoa protein, H15LP: 15 min hydrolyzed lentil protein, and H60LP: 60 min hydrolyzed lentil protein.

**Table 7:** Multifactorial ANOVA on the encapsulation efficiency of total polyphenols and bixin present in annatto extract.

Parameter	EE (%)
Principal effects	*p*-value
A: Structural modification	≤0.001
B: Bioactive compounds encapsulated	≤0.001
C: Protein source	≤0.001
AB	0.053
AC	0.006
BC	0.001

## Data Availability

The original contributions presented in the study are included in the article, further inquiries can be directed to the corresponding authors.
